# Molecular Disorder of Bicalutamide—Amorphous Solid Dispersions Obtained by Solvent Methods

**DOI:** 10.3390/pharmaceutics10040194

**Published:** 2018-10-18

**Authors:** Joanna Szafraniec, Agata Antosik, Justyna Knapik-Kowalczuk, Karolina Gawlak, Mateusz Kurek, Jakub Szlęk, Witold Jamróz, Marian Paluch, Renata Jachowicz

**Affiliations:** 1 Department of Pharmaceutical Technology and Biopharmaceutics, Faculty of Pharmacy, Jagiellonian University Medical College, Medyczna 9, 30-688 Krakow, Poland; agata.antosik@uj.edu.pl (A.A.); mateusz.kurek@uj.edu.pl (M.K.); j.szlek@uj.edu.pl (J.S.); mfjamroz@cyf-kr.edu.pl (W.J.); mfjachow@cyf-kr.edu.pl (R.J.); 2 Department of Physical Chemistry and Electrochemistry, Faculty of Chemistry, Jagiellonian University, Gronostajowa 2, 30-387 Krakow, Poland; gawlak@chemia.uj.edu.pl; 3 Division of Biophysics and Molecular Physics, Institute of Physics, University of Silesia, Uniwersytecka 4, 40-007 Katowice, Poland; justyna.knapik-kowalczuk@smcebi.edu.pl (J.K.-K.); marian.paluch@us.edu.pl (M.P.); 4 Silesian Center for Education and Interdisciplinary Research, 75 Pulku Piechoty 1a, 41-500 Chorzow, Poland

**Keywords:** bicalutamide, PVP, solid dispersion, spray drying, evaporation, dissolution, amorphization

## Abstract

The effect of solvent removal techniques on phase transition, physical stability and dissolution of bicalutamide from solid dispersions containing polyvinylpyrrolidone (PVP) as a carrier was investigated. A spray dryer and a rotavapor were applied to obtain binary systems containing either 50% or 66% of the drug. Applied techniques led to the formation of amorphous solid dispersions as confirmed by X-ray powder diffractometry and differential scanning calorimetry. Moreover, solid–solid transition from polymorphic form I to form II was observed for bicalutamide spray dried without a carrier. The presence of intermolecular interactions between the drug and polymer molecules, which provides the stabilization of molecularly disordered bicalutamide, was analyzed using infrared spectroscopy. Spectral changes within the region characteristic for amide vibrations suggested that the amide form of crystalline bicalutamide was replaced by a less stable imidic one, characteristic of an amorphous drug. Applied processes also resulted in changes of particle geometry and size as confirmed by scanning electron microscopy and laser diffraction measurements, however they did not affect the dissolution significantly as confirmed by intrinsic dissolution study. The enhancement of apparent solubility and dissolution were assigned mostly to the loss of molecular arrangement by drug molecules. Performed statistical analysis indicated that the presence of PVP reduces the mean dissolution time and improve the dissolution efficiency. Although the dissolution was equally affected by both applied methods of solid dispersion manufacturing, spray drying provides better control of particle size and morphology as well as a lower tendency for recrystallization of amorphous solid dispersions.

## 1. Introduction

The amorphization of active pharmaceutical ingredients (APIs) and the characterization of the properties of molecularly disordered solid-state have attracted attention in recent years. Its formation occurs when a molten crystalline substance is rapidly cooled down as the molecules have not had enough time to move from their current position to a thermodynamically stable position on the crystal lattice and to rearrange into an ordered system [1]. The formed supercooled liquid state remains in equilibrium with the molten substance until reaching the glass transition temperature (*T*_g_), at which a glassy state exhibiting only short range order is achieved. This second order thermodynamic transition is associated with an increase in enthalpy, entropy, volume and free energy of the amorphous state in comparison to its crystalline counterpart [2,3].

Although the crystalline state offers the advantages of good chemical and physical stability as well as high purity, the lattice energy barrier often affects drug solubility. The absence of the long-range order leads to higher molecular mobility of the system which enables it to reach supersaturation and consequently a higher overall dissolution rate. It is of particular importance in the drug development as approximately 46% of APIs fall into class II of the biopharmaceutical classification system due to poor solubility in water and dissolution rate-limited oral absorption [4].

The increase in apparent solubility of amorphous drugs is an advantage, however the inherent stability remains as a major limitation. The systems tend to recrystallize upon introduction to the aqueous environment, either through precipitation from the supersaturated solution during the dissolution process or upon storage through contact with moisture in the air. The nucleation and the crystallization of amorphous material is accelerated above the *T*_g_ due to the increased mobility of the disordered system; thus, it is postulated to assess the molecular dynamics of the molecular system and to manufacture compounds with a high glass transition temperature. It is particularly challenging for drugs with low *T*_g_ (e.g., paracetamol) as the excess free energy leads to spontaneous transformation to the crystalline state. Thus, drug-polymer systems have been introduced as they show improved stability in comparison to amorphous drugs alone [5].

Due to their complex three-dimensional (3D) architecture, macromolecules hinder the molecular mobility of amorphous systems, lowering their chemical potential and preventing devitrification. The formation of solid dispersions with polymers having high *T*_g_ values leads to the antiplasticization of the API molecules which is manifested by the increase in *T*_g_ combined with the increase of free energy required for crystallization. The glass transition of the mixture can be calculated by use of the Gordon–Taylor equation [6]. Other factors which drive the formation of amorphous solid dispersions exhibiting enhanced dissolution are the interactions between drug and polymer molecules as well as the type and parameters of the applied process. 

The term solid dispersion describes the systems in which one or more APIs are molecularly dispersed within an inert carrier. The two main manufacturing techniques rely on melting and evaporating the solvent [7]. In the fusion method, the heating of a mixture of the drug and polymer is followed by fast cooling that leads to solidification of the molten system. Although this method is very simple and frequently used, it faces several limitations resulting from the limited miscibility of APIs and the carriers, as well as the thermal lability of drug molecules [8]. The solvent-based methods solve these problems as the drug and the carrier are dissolved in a volatile solvent such as ethanol, ethyl acetate, methanol or an ethanol–methylene chloride mixture which can be removed without heating [9,10]. Laboratory solvent evaporation techniques can be divided into several groups such as rotary evaporation, spray drying and freeze drying. In a rotary evaporator, the solvent is removed under vacuum at an only slightly elevated temperature which prevents the decomposition of the drug or the carrier. Although the complete removal of the solvent is almost impossible and residual solvent may cause toxicity and plasticize the system leading to phase separation, the method has been successfully applied to the preparation of solid dispersions containing celocoxib [11], nifedipine [12], itraconazole [13] and stiripentol [14]. 

The spray drying process is a very efficient technique for manufacturing solid dispersions as it provides rapid solvent removal and fast formation of solid API-carrier particles [15]. Moreover, it is a scalable technique that provides the possibility of continuous manufacturing, good uniformity of molecular dispersion and a low production cost on an industrial scale [7]. It has been described in the manufacturing of solid dispersions containing celocoxib [16], tadalafil [17], itraconazole [18] and glibenclamide [19]. Moreover, spray drying was used for the preparation of marketed products such as Incivek^®^ and Intelence^®^ [20]. This technique comprises of four processes: the atomization of the liquid with the dissolved or suspended drug, which is transported into the nozzle and sprayed onto fine droplets; the mixing of the liquid with the drying gas; and the evaporation and separation of obtained particles from the gas by the use of cyclone [21]. The size of the droplets can be modulated via the nozzle and thus spray drying is used as a technique for particle engineering, leading to particle size reduction. Moreover, this technique provides amorphization of APIs which results in significant improvement in their solubility and dissolution rate. 

The results reported herein describe the amorphization of bicalutamide (BCL)—a non-steroidal antiandrogenic drug used in prostate cancer treatment. This poorly-soluble compound is assigned to BCL class II due to its low solubility in water (<3.7 mg/L) and high membrane permeability (logP = 2.92) [22]. Moreover, it has two polymorphic forms and easily undergoes mechanical activation [23,24,25,26]. In our research, we applied a rotary evaporator and spray dryer in order to obtain solid dispersions containing an equal amount of bicalutamide and polyvinylpyrrolidone (PVP) as well as the system with an excess of the carrier. The effect of the applied process on phase transition, physical stability, saturation solubility and the dissolution kinetics of BCL was investigated. X-ray diffraction measurements, differential scanning calorimetry and infrared spectroscopy were applied to study process-induced phase transitions of the drug. The physical stability of the amorphous solid dispersions were analyzed as well. The morphology and particle size distribution were assessed by scanning electron microscopy and laser diffraction. 

## 2. Materials and Methods 

### 2.1. Materials

Bicalutamide (BCL, *N*-[4-cyano-3-(trifluoromethyl)phenyl]-3-[(4-fluorophenyl)sulfonyl]-2-hydroxy-2-methylpropanamide, 99.8%, Hangzhou Hyper Chemicals Limited, Zhejiang, China) was used as the model drug. Polyvinylpyrrolidone K29/32 (PVP, Ashland, Covington, KY, USA) was used as an excipient. Sodium lauryl sulfate (SLS, BASF, Ludwigshafen am Rhein, Germany) was used to prepare the dissolution medium. Ethanol (absolute, 99.8%, pure p.a., Avantor Performance Materials, Gliwice, Poland) was used as a solvent in evaporation processes. Cyclohexane (ACS, pure p.a., Avantor Performance Materials, Gliwice, Poland) was used as a dispersant in laser diffraction measurements. All chemicals were used as received. Distilled water was used to prepare all of the aqueous solutions.

### 2.2. Solvent Evaporation 

Bicalutamide (1.5 g) was dissolved in 200 mL of ethanol and mixed with PVP at a 1:1 and 2:1 weight (wt.) ratio, respectively. The solution was heated up to 40 °C in a water bath and after complete dissolution of the mixture, the solvent was evaporated under reduced pressure using a Hei-VAP Value rotavapor (Heidolph, Schwabach, Germany). The rotational speed was 200 rpm and the pressure was reduced stepwise to approximately 50 mbar. Obtained samples were stored under vacuum for 24 h prior further characterization. 

### 2.3. Spray Drying 

Ethanolic solutions containing bicalutamide and PVP were obtained similarly to those for solvent evaporation and spray dried using a Mini Spray Dryer B-191 (Büchi, Flawil, Switzerland). The process was conducted using the following parameters: inlet temperature = 50 °C, outlet temperature = 42 °C, aspirator flow = 100%, gas flow rate = 600 L/min, liquid flow rate = 3.4 mL/min, diameter of nozzle = 0.7 mm. The process was carried out under constant control and the concentration of ethanol was 10 times lower than the flammability limit. The samples were further dried under vacuum to remove residual solvent. The yields were approximately 60–70%.

### 2.4. Scanning Electron Microscopy (SEM)

The morphological features of samples were determined using a Phenom Pro desktop electron microscope (PhenomWorld, Thermo Fisher Scientific, Waltham, MA, USA) equipped with a CeB6 electron source and a backscattered electron detector. The powder was placed on the conductive adhesive tape previously glued to the specimen mount. The sample excess (loosely bound to the tape) was removed using a stream of argon. The acceleration voltage was equal to 10 kV and the magnification was 500× and 1000× for evaporated samples, and 5000× for spray dried systems. The measurements were performed without further processing.

### 2.5. Differential Scanning Calorimetry (DSC) 

The thermodynamic properties of the samples were examined using a DSC 1 STAR^e^ System (Mettler–Toledo, Greifensee, Switzerland) equipped with a HSS8 ceramic sensor with 120 thermocouples and a liquid nitrogen cooling station. The measuring device was calibrated for temperature and enthalpy using zinc and indium standards. Crystallization and melting points were determined as the onset of the peak, whereas the glass transition temperature was determined as the midpoint of the heat capacity increment. The samples were measured in an aluminum crucible (40 μL). All measurements were carried out with the heating rate equal to 10 K/min.

### 2.6. Powder X-ray Diffraction (PXRD)

The diffraction patterns of the samples were registered at ambient temperature within the angular range of 3–70° using a Mini Flex II X-ray diffractometer (Rigaku, Tokyo, Japan) with 5°/min step of size equal to 0.02. Monochromatic Cu Kα radiation (λ = 1.5418 Å) was used.

### 2.7. Laser Diffraction Measurements

Particle size distribution was determined using a Mastersizer 3000 (Malvern Instruments, Malvern, UK) equipped with a HydroEV unit. Samples were analyzed by the wet method using cyclohexane (refractive index, RI = 1.426) as a dispersant. Fraunhofer diffraction theory was applied to find the relationship between particle size and the light intensity distribution pattern. Reported data represents the averages from ten series of measurements of each sample.

### 2.8. Infrared Spectroscopy (FTIR)

A Nicolet iS10 FT-IR spectrometer (Thermo Fisher Scientific, Waltham, MA, USA) equipped with the Smart iTR™ ATR sampling accessory with diamond as the ATR crystal was used. Spectra of powders were collected within the range 600–4000 cm^−1^ with 4 cm^−1^ resolution, 128 scans for each sample.

### 2.9. Dissolution Study

The dissolution of BCL was determined using the method recommended by the FDA for BCL tablets (1000 mL of 1% SLS, 50 rpm, 37 ± 0.5 °C) in the pharmacopeial paddle dissolution apparatus Vision Elite 8 (Hanson Research, Chatsworth, CA, USA) equipped with VisionG2 AutoPlus Autosampler. Pure drug and binary systems, equivalent to 50 mg of BCL, were placed into the beakers. The sink conditions were maintained. The samples were filtered and analyzed at 272 nm at predetermined periods of time using a UV-1800 spectrophotometer (Shimatzu, Kioto, Japan) equipped with flow-through cuvettes. The tests were carried out in triplicate and presented results represent averages with their standard deviations (mean ± SD).

### 2.10. Intrinsic Dissolution Rate Study

The compacts for intrinsic dissolution studies were prepared using a Specac 50 hydraulic press (Specac, Kent, UK) by compressing powder systems directly into the stainless steel cylinders with a 0.5026 cm^2^ flat surface area and 2 tons of pressure applied for 30 s. The cylinders were then mounted in the Hanson Vision G2 Elite 8 dissolution apparatus (Hanson Research, Chatsworth, California, USA) and stirred with a rotation speed of 100 rpm. The dissolution vessels were filled with 500 mL of 1% SLS solution maintained at 37 °C. The amount of dissolved BCL was assayed online using a UV-1800 spectrophotometer (Shimadzu, Kioto, Japan) at λ = 272 nm. The intrinsic dissolution rate was calculated based on the accessible surface area as a function of mass ratio of the components in the mixture. Ten points were used for the construction of intrinsic dissolution profiles of each system and four of them were used to calculate the intrinsic dissolution rate (IDR) values.

### 2.11. Solubility Study

An excess of processed bicalutamide or BCL–PVP binary systems of both type (i.e., evaporated and spray dried) was dispersed in 25 mL of distilled water. The suspensions were shaken at room temperature for 24 h using the KS 130 basic orbital shaker (IKA, Staufen im Breisgau, Germany). The samples were centrifuged at 3600 rpm for 20 min in the MPW 221 apparatus (MPW MED Instruments, Warsaw, Poland) and filtered through a 0.45 µm Chromafil^®^Xtra CA-45/25 membrane filter. The samples were assayed in triplicate at λ = 270 nm using a UV-1800spectrophotometer (Shimadzu, Kioto, Japan). The reported data represents the averages from three series of measurements with standard deviations (mean ± SD).

### 2.12. Statistical Analysis 

The KinetDS v3.0 software was used to determine the dissolution kinetics of BCL. Three mathematical models (zero order, Korsmeyer–Peppas, and Hixson–Crowell) were fitted to the individual dissolution data. To assess the goodness of fit, the coefficient of determination was calculated in combination with the analysis of variance (alpha < 0.01). Moreover, the models’ independent parameters, such as dissolution efficiency (DE) and mean dissolution time (MDT) were also calculated.

## 3. Results and Discussion

### 3.1. Molecular Arrangement

#### 3.1.1. X-ray Powder Diffraction Studies

**Figure 1:**
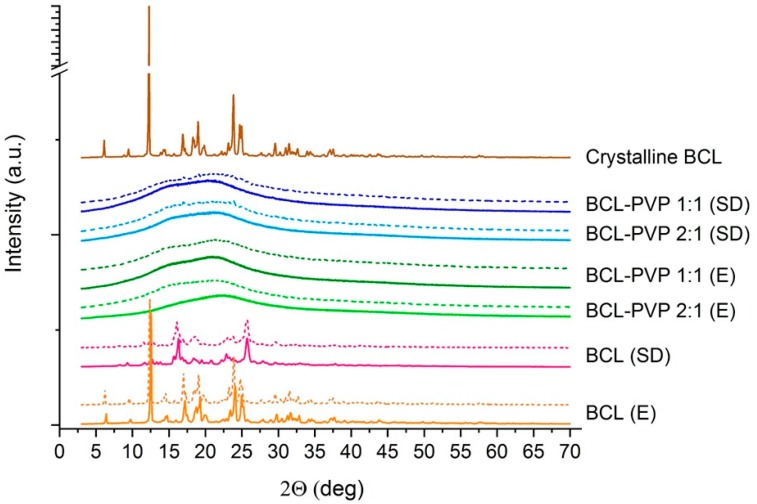
X-ray diffraction patterns of crystalline bicalutamide (BCL) and BCL–PVP (polyvinylpyrrolidone) binary systems obtained by either evaporation (E) or spray drying (SD) measured two days (solid lines) and six months (dotted lines) after preparation.

The molecular structure of solid dispersions and bicalutamide processed using either a rotavapor or a spray dryer indicates that the drug undergoes structural changes (Figure 1). The sharp Braggs peaks in the diffractogram registered for raw BCL confirm that bicalutamide exists as a form I polymorph [27]. The long-range order was not affected in the evaporation process as the diffraction pattern is almost unchanged with only a slight decrease in the relative intensity of peaks in the range of 15–25°. This indicates the recrystallization of BCL during the solidification and the reconstruction of the monoclinic crystal lattice characteristic for form I polymorphs. Interestingly, there were different Braggs peaks of bicalutamide after spray drying and the diffraction pattern was less structured than the one obtained for the evaporated sample. The peaks can be assigned to polymorphic form II, which indicates that spray drying of bicalutamide led to phase transition. Moreover, obtained results indicate that storing samples for six months did not lead to structural changes of either evaporated or spray dried samples (dotted lined in Figure 1). What is worth mentioning is that form II is metastable and it usually undergoes solid–solid transition into the more stable form I [28]. Results presented in Figure 1 indicate that spray dried bicalutamide exists as a form II polymorph even after six months when stored at ambient temperature. No shifts or peak displacement were noticed in the diffraction pattern. 

The samples containing PVP were found to be totally amorphous as no structured diffraction patterns were noticed. The broad halo confirms that the molecular structure of binary systems is disordered and that PVP sufficiently prevents the recrystallization of bicalutamide from both evaporated and spray dried systems. The samples containing a two-fold excess of the drug in comparison to the mass of the carrier did not differ from those containing an equal mass of BCL and PVP. This is particularly important for further applications as it may reduce the amount of auxiliary substance in the final dosage form. It also confirms that PVP serves as a good stabilizer for supercooled BCL and the excess of the carrier is not obligatory. It stays in contradiction with previously published papers where the concentration of PVP providing stabilization for the amorphous form of bicalutamide exceeded 80% [29,30]. In performed studies, both types of systems (i.e., containing either 50% or 33% of the carrier) were totally amorphous. After six months, broad halos were registered for all binary systems and no signs of recrystallization were noticed. This is of particular importance as molecularly-disordered BCL usually recrystallizes into a form II polymorph within days and further into the most stable form I. The addition of PVP prevented the reconstruction of the crystal lattice. Importantly, research focused on the physical stability of the glassy state of bicalutamide confirmed that PVP hindered recrystallization of the drug. Szczurek et al. applied broadband dielectric spectroscopy and estimated the physical stability of amorphous bicalutamide would be approximately 145 years for a BCL–PVP 2:1 binary system when stored at room temperature [31]. Humidity effect was not considered, which makes that estimation more theoretical. 

#### 3.1.2. DSC Studies

**Figure 2:**
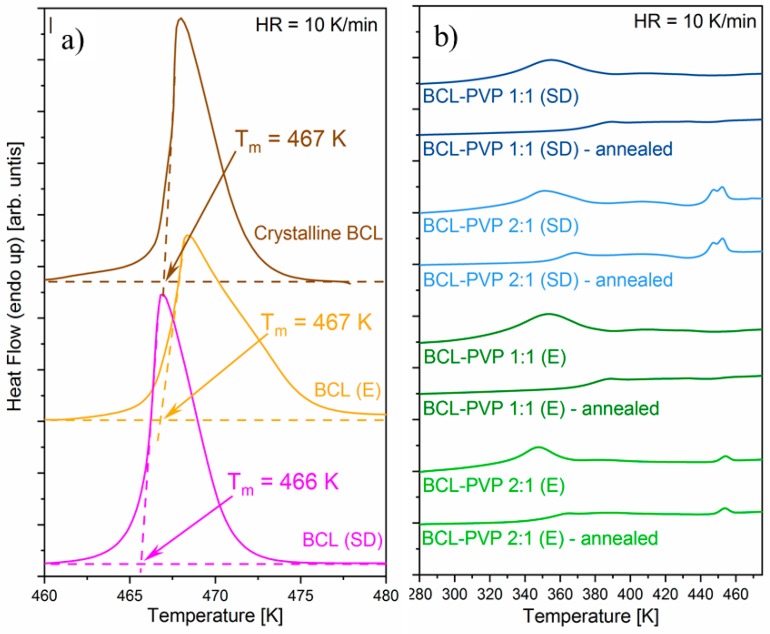
The DSC thermograms of binary systems containing bicalutamide alone (**a**) and BCL–PVP solid dispersions (**b**).

Thermal properties of BCL and BCL–PVP solid dispersions obtained by either spray dryer or rotavapor have been investigated using the DSC technique. The samples were heated from 273 K to 483 K at a rate of 10 K/min. The DSC thermograms of neat BCL prepared by employing two different methods are compared to crystalline samples (as purchased) and presented in Figure 2a. As can be seen, none of the applied methods led to BCL amorphization. On the DSC thermograms, one can observe the endothermic peak in the vicinity of 470 K, corresponding to the drug melting. It is worth highlighting that even if the amorphization had failed, some differences between evaporated and spray dried BCL exist. In the DSC thermogram of the spray dried sample, one can observe that the position of the endothermic peak is slightly shifted to a lower temperature in comparison to the initial BCL, which may indicate the formation of a type II polymorph. In the case of the evaporated BCL, the melting peak is at the same position as in the case of the initial sample. 

Since either spray drying or evaporation methods were not able to convert the neat BCL to its amorphous form, samples containing PVP were prepared accordingly. The DSC thermograms of all obtained solid dispersions are presented in Figure 2b. For each sample, two separate experiments were performed. During the first experiment, the sample was heated from 273 K to 483 K at a rate of 10 K/min as in the case of neat BCL. The second experiment consisted of three segments: (i) 15-min annealing of the sample at 353 K, (ii) cooling the sample to 273 K at a rate of 10 K/min and (iii) heating the sample in the same way as in the case of the first experiment. As can be seen, all DSC thermograms obtained without annealing of BCL–PVP solid dispersions are characterized by the broad endothermic peak in the vicinity of 340–380 K. Since this peak vanished after annealing, one might conclude that it reflects the water evaporation. The DSC thermograms of BCL–PVP 1:1 obtained after the annealing procedure are characterized by a single step-like glass transition thermal event. On the thermogram of the BCL–PVP 2:1 systems, next to a glass transition event, an exothermic peak representing cold crystallization followed by endothermic peak reflecting the melting of BCL can be observed. The temperatures taken as the midpoint of the samples glass transition and the onsets of cold crystallization and melting are shown in Table 1. The presence of either *T*_g_ or T_c_ indicates that all samples containing drug and polymer were amorphous. Obtained results shows that the larger the amount of PVP, the higher the values of *T*_g_. The shift in the glass transition temperature, as well as the lack of cold crystallization in BCL–PVP 1:1, indicates that such a system should be more physically stable than BCL–PVP 2:1 when stored at standard storage conditions. Due to the fact that the BCL–PVP 2:1 system prepared by evaporation re-crystallizes at a lower temperature, it is characterized by lower physical stability than its spray dried counterpart. Consequently, it can be concluded that spray drying is the better method for the production of BCL-based amorphous solid dispersions.

#### 3.1.3. Infrared Spectroscopy

**Figure 3:**
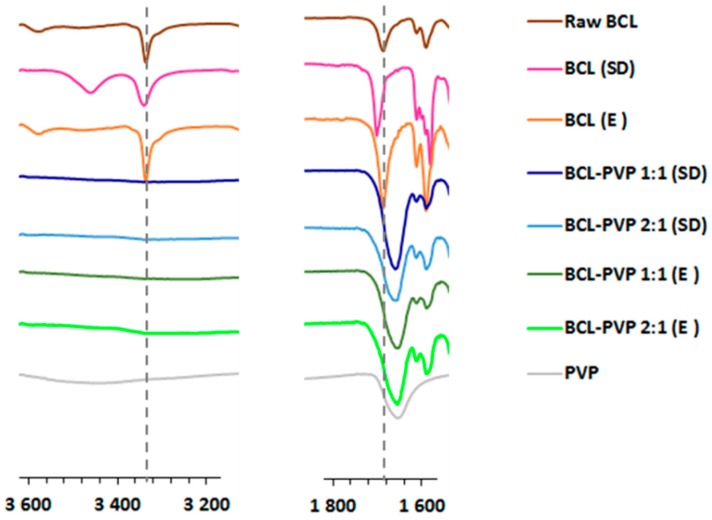
The comparison of FTIR spectra of raw bicalutamide, drug processed without a carrier and BCL–PVP solid dispersions.

FTIR studies were used to analyze the interactions between bicalutamide co-processed with PVP, as they may be important factors for the stabilization of amorphous solid dispersion (Figure 3). Moreover, spectroscopic characteristics of the binary systems, i.e., peak intensity, shape, width and position, can be used to distinguish polymorphs and amorphous forms of the compound. The spectrum of raw BCL abounds in peaks characteristic to polymorphic form I [27] with well-structured peaks of the stretching vibrations of functional groups, i.e., C=O at 1687 cm^−1^, N-H at 3336 cm^−1^, O-H between 3558–3609 cm^−1^ and C≡O at 1326 cm^−1^ [23]. Solvent removal in a rotavapor did not cause significant changes in the spectrum of bicalutamide processed without a carrier. A shift of the CO absorption band towards higher wavenumbers was observed only for spray dried drug, which indicates the formation of a form II polymorph [27]. The differences were well pronounced for systems containing bicalutamide and PVP. They were most visible in two spectral regions: 1600–1800 cm^−1^ and 3200–3600 cm^−1^. The shift and the broadening of the band corresponding to C=O stretching vibration in binary systems indicates that the components of solid dispersion interact intermolecularly, which can be responsible for the stabilization of molecularly disordered systems as confirmed by XRPD and DSC measurements. Moreover, the band at 3336 cm^−1^ almost disappeared, which suggests that the amide form of crystalline bicalutamide was replaced by a less stable imidic one characteristic of an amorphous drug [32].

### 3.2. Particle Size and Morphology

#### 3.2.1. Morphology of Particles from Scanning Electron Microscopy

**Figure 4:**
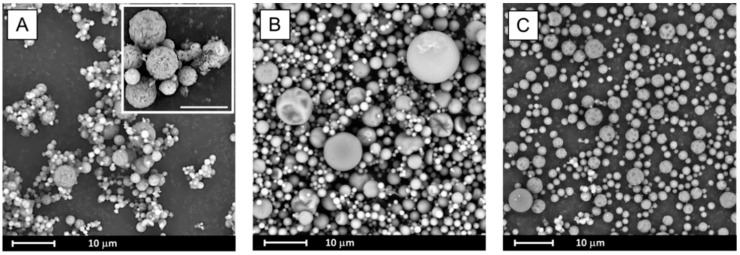
Scanning electron microscopy (SEM) images of spray dried systems: BCL (**A**), BCL–PVP 2:1 (**B**) and BCL–PVP 1:1 (**C**). Scale bar in the insert is 5 µm.

SEM analysis indicates that spray drying led to significant morphological changes of processed samples as seen in Figure 4. The formation of spherical particles was observed. Their diameter did not exceed 10 µm for BCL spray dried alone (Figure 4A) and with PVP (1:1 wt. ratio, Figure 4C) and 15 µm for BCL spray dried with PVP at a 2:1 wt. ratio (Figure 4B). While particles of the drug co-processed with PVP are smooth, the surface of BCL spray dried particles seems to be more rough (they look like cotton-balls, see insert in Figure 4A).

**Figure 5:**
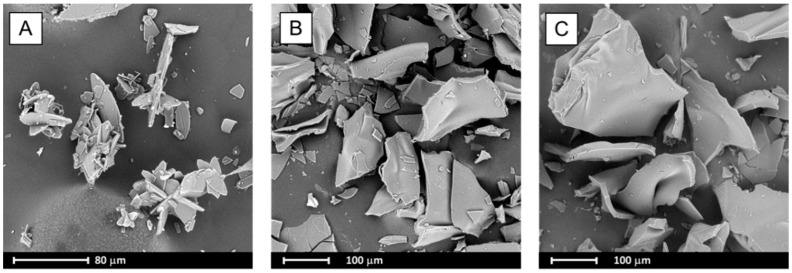
SEM images of evaporated systems: BCL (**A**), BCL–PVP 2:1 (**B**) and BCL–PVP 1:1 (**C**).

During the evaporation process, bicalutamide undergoes recrystallization and a reduction in particle size was observed. Formed crystals adopt a plate-like shape and tend to aggregate into agglomerates not exceeding 120 µm in length (Figure 5A), whilst for raw BCL, elongated hexagons of approximately 150 µm in length were observed [23]. Particles obtained after solvent removal from systems containing BCL and PVP also adopt plate-like shapes, however they are bigger (exceed 200 µm) (Figure 5B,C).

#### 3.2.2. Particle Size Distribution from Laser Diffraction Measurements

Data obtained from SEM analysis corresponds well with particle size determination utilizing laser diffraction (Table 2). Spray drying led to the formation of particles not exceeding 70 µm in diameter, while the particles of evaporated samples are greater than 200 µm for solid dispersions and 50 µm for bicalutamide processed without a carrier.

**Figure 6:**
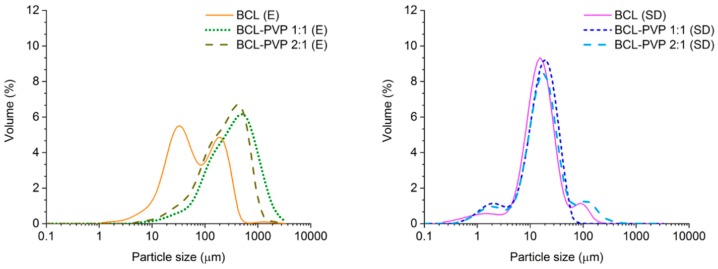
Particle size distribution of evaporated (E) and spray dried (SD) bicalutamide and BCL–PVP binary systems (1:1 and 2:1 weight ratio, respectively).

The obtained particle size distribution of evaporated samples is monomodal (BCL–PVP systems), broad and slightly tailed towards low particle size (Figure 6). It correlates with previously described SEM data where particles of various sizes were observed. Interestingly, the distribution of particles of BCL after solvent removal by means of evaporation exhibits two maxima, one at approximately 30 µm and one at 200 µm. This may be due to an aggregation of small plate-like particles into a bigger one. Moreover, the distribution is shifted towards lower size values in comparison to those collected for co-processed samples. The size distributions of spray dried systems confirm the formation of much smaller particles than after evaporation (Figure 6). It is clearly visible that there are particles of a diameter below 1 µm (in each system), which may affect the equilibrium solubility. The maxima of the distributions of particles obtained by spray drying are located within a similar range, below 20 µm.

### 3.3. Dissolution Study

Papers published so far reported formation of amorphous bicalutamide by means of the formation of solid dispersion with PVP utilizing solvent evaporation; however, an excessive amount of a carrier was required [29,30]. The drug was only totally amorphous when the content of the polymer exceeded 80%. This was concluded as a disadvantage in further formulation studies as it may lead to an increase in the bulkiness and weight of the tablets.

**Figure 7:**
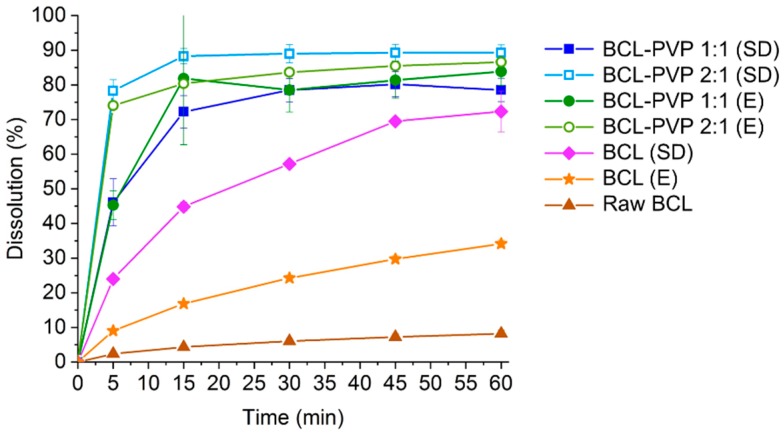
Dissolution of crystalline bicalutamide, drug processed alone and binary systems containing bicalutamide and PVP (1:1 and 2:1 weight ratio, respectively) obtained using evaporation technique (E) and spray drying (SD).

To face that limitation, we prepared BCL–PVP binary systems containing either an equal amount of both substances or twice as much BCL as the carrier. The dissolution profiles presented in Figure 7 showed approximately 10-fold improvement in bicalutamide dissolution from solid dispersions in comparison to the untreated drug where only 8.2% of the API dissolved. This may be explained by drug amorphization as molecularly disordered systems exhibit improved dissolution. Interestingly, while the amount of drug dissolved from evaporated samples does not differ significantly between the two investigated systems (less than 3%, i.e., 83.85 ± 4.04% for 1:1 solid dispersion and 86.59 ± 1.21% for the 2:1 binary system), variations between spray dried systems are more pronounced (approximately 10%, i.e., 78.51 ± 3.36% for the 1:1 system and 89.29 ± 2.27% for 2:1 solid dispersion). However, both of the applied methods lead to similar dissolution profiles and the total amount of dissolved drug indicates that the dissolution behavior is affected strongly by the molecular characteristic of the sample (the excess of free energy possessed by the amorphous state). The effect of different size and morphology of solid-state particles is less significant. The phase transition from polymorphic form I to form II resulted in an eight-fold improvement of spray dried bicalutamide dissolution as 72.33 ± 5.91% of drug dissolved. As there were no differences in particle morphology and size between bicalutamide spray dried alone and with PVP, we assumed that the effect of particle size reduction is of minor importance. Importantly, evaporation did not result in structural changes of the drug molecule and the four-fold improvement in bicalutamide dissolution (34.14 ± 1.05% after 1 h) was concluded to be the result of particle size reduction and slightly decreased crystallinity of the sample in comparison to raw BCL. Regardless of the method of preparation of solid dispersion, more of the drug dissolved from the BCL–PVP 2:1 binary system. It confirms that the excess of PVP is not required for either BCL dissolution enhancement or for stabilization of the amorphous form of the drug.

### 3.4. Saturation Solubility

**Figure 8:**
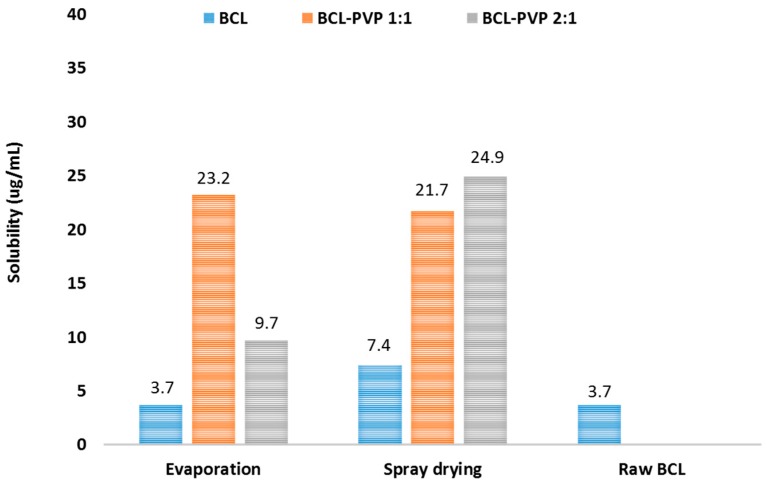
Solubility of bicalutamide and BCL–PVP binary systems obtained using evaporation technique and spray drying.

The solubility of bicalutamide spray dried alone is twice as much as the unprocessed drug. This may be a result of phase transition from polymorph I to polymorph II, which exhibits over two-fold higher solubility. Observed improvement can also be a consequence of particle size reduction (Figure 8). While the solubility theory suggests that the effect of particle size reduction on equilibrium solubility is slightly noticeable, the Ostwald–Freundlich equation indicates that the relative increase in compound activity (and thus solubility) related to particle size reduction becomes significant below 1 µm. This assumption was adopted from the phenomena described by Kelvin for gas–liquid systems where the increased vapor pressure and liquid transfer into the gas phase was related to increased curvature (decreased radii) of the liquid droplets [33]. In solid–liquid systems, the reduction of particle size affects neither the efficiency of solvation nor the properties of the solid state, however below 1 µm, the solvation pressure increases, causing an increase in solubility [34]. According to SEM images and particle size distribution from laser diffraction measurements, there is a fraction of particles with a diameter below 1 µm; this could be responsible for the observed increase in solubility of bicalutamide spray dried without a carrier. No improvement in evaporated bicalutamide was noticed.

For systems co-processed with PVP, the increase in solubility varied between 2.6-fold for the 2:1 evaporated system to 6.7-fold for the 2:1 spray dried mixture. No significant variations between spray dried samples were observed and similarly to dissolution tests, a better result was obtained for the 2:1 system. Interestingly, evaporated samples varied in saturation solubility as the value determined for 1:1 solid dispersion was two times greater than for the 2:1 binary system. This conforms with the DSC data, as evaporated samples, especially those containing an excess of the drug, tend to recrystallize faster than spray dried ones.

### 3.5. Intrinsic Dissolution Study

The intrinsic dissolution measurements have been recently studied and discussed as an important tool in pharmaceutical research and development. It allows the characterization of the crystalline forms and polymorphs of pure drugs and drug formulations by exposing a constant surface area to the dissolution medium. It has been also introduced for the solubility determination as an alternative method to equilibrium solubility [35,36]. 

**Figure 9:**
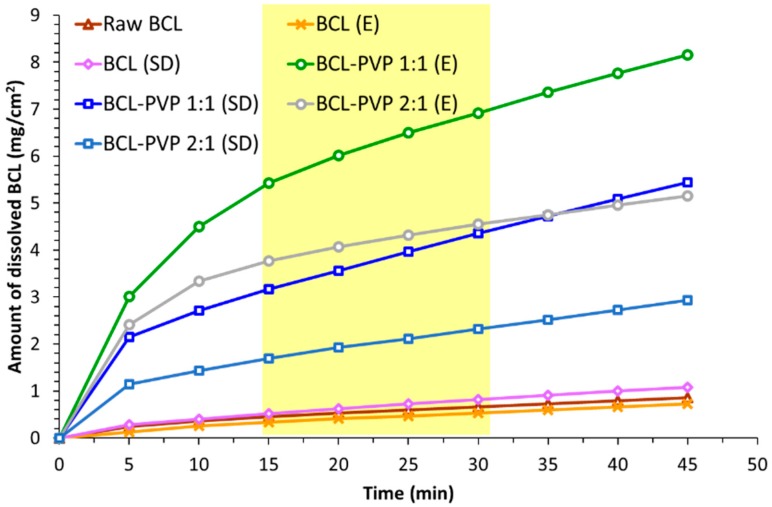
Intrinsic dissolution of crystalline bicalutamide, drug processed alone and binary systems containing bicalutamide and PVP obtained using the evaporation technique (E) and spray drying (SD). Yellow square indicates the range used to calculate the intrinsic dissolution rate (IRD) values.

Performed intrinsic dissolution studies showed significant differences in the dissolution behavior that is not affected by either particle size distribution or porosity (Figure 9). In case of evaporated binary systems, compacts underwent erosion and the presence of freely floating particles in the dissolution medium might cause slightly enhanced dissolution and consequently overestimated values of IDR. The observed behavior of compacts may be a result of the plate-like shape of the particles and indicates poor tabletability of this system.

The intrinsic dissolution rate (IDR) values presented in Table 3 clearly confirm that faster dissolution was obtained for the systems prepared by spray drying. It can be also noticed that a 1:1 concentration of BCL and PVP promotes faster dissolution in both preparation methods as the IDR for these samples was higher in comparison to 2:1 binary systems. This may result from the faster dissolution of the hydrophilic carrier and increase of the BCL surface available for the solvent. It can also be concluded that the processing of bicalutamide itself in a rotary evaporator did not affect the intrinsic dissolution rate significantly, while spray drying slightly increases the intrinsic dissolution rate.

### 3.6. Statistical Analysis

The drug dissolution mechanism was explained by fitting the zero order, Korsmeyer–Peppas, and Hixson–Crowell mathematical models (Table 4) using the KinetDS v3.0 software [37,38]. The highest coefficients of determination (r^2^) were found for Korsmeyer–Peppas models. Considering the coefficients of determination (r^2^) and the significance of ANOVA tests, three formulations failed to fit the chosen models (BCL-PVP 1:1 (SD), BCL-PVP 2:1 (SD) and BCL-PVP 1:1 (E)). Fitted Korsmeyer–Peppas models for pure BCL (processed or not) indicate that the dissolution from these formulations is diffusion-driven and may be connected to the crystalline or recrystallized form (Table 5). Moreover, the BCL-PVP 2:1 (E) formulation seems to follow diffusion-based kinetics after the first stage of fast dissolution. Although the fitting of the models failed for spray dried formulations consisting of BCL and PVP, the highest values of the coefficient of determination for the Korsmeyer–Peppas model may suggest that bicalutamide can be dissolved by diffusion (r^2^ = 0.7916–0.8413) rather than by the erosion-based mechanism (r^2^ = 0.5014–0.5611) or the zero order kinetics (r^2^ = 0.5043–0.5796).

In general, according to the calculated model independent parameters, it is observed that the addition of PVP reduces the mean dissolution time (MDT) by two- to three-fold. The shortest MDT was obtained for a formulation prepared by the spray drying technique (BCL-PVP 2:1). The longest MDT was observed for BCL alone prepared by the evaporation technique. Therefore, PVP increases the dissolution efficiency by three-fold in case of evaporation processing and by 1.5-fold for the spray dried samples in comparison to drug processed alone.

## 4. Conclusions

In this paper, we analyzed the effect of solvent evaporation techniques on physical stability and dissolution of bicalutamide from solid dispersions containing PVP as a carrier. Obtained results indicate that both applied methods, namely spray drying and evaporation utilizing a rotary evaporator, led to amorphization of bicalutamide as confirmed by X-ray diffractometry and DSC measurements. The presence of intermolecular interactions between the drug and polymer confirmed by FTIR spectroscopy was found to provide stabilization of the molecularly-disordered system. Importantly, the concentration of PVP that provided sufficient stabilization of amorphous bicalutamide was 33% wt. while the previously published papers reported as much as a five-fold excess of the carrier. 

The loss of molecular arrangement by drug molecules led to its enhanced dissolution. Over a 10-fold improvement was noticed in comparison to the untreated drug. Moreover, the solubility of solid dispersions in water was also improved from 3.7 mg/L to as much as 24.9 mg/L. Interestingly, the solubility of bicalutamide spray dried without PVP led to a two-fold enhancement which resulted from phase transition into a form II polymorph. Statistical analysis confirmed that addition of PVP led to the increase of dissolution efficiency and significant decrease of mean dissolution time in comparison to raw bicalutamide and the drug processed alone.

The removal of solvent led to morphological changes in the particles. Bicalutamide polymorphic form I that crystallizes in a monoclinic lattice system appears as elongated hexagons, while spray drying (with or without PVP) led to the formation of spherical particles with a diameter below 15 µm. Applying the rotary evaporator resulted in the formation of plate-like particles of irregular shape exceeding 100 µm and exhibiting a wide size distribution. While the dissolution was equally affected by both applied methods of solid dispersion manufacturing, spray drying provided better control of particle size and morphology as well as a lower tendency for recrystallization of amorphous solid dispersions. Thus, it is more useful in the pharmaceutical industry.

## Figures and Tables

**Table 1:** Comparison of the glass transition temperature (*T*_g_), *T*_c_ and *T*_m_ values of binary systems containing BCL and PVP.

Process	Sample	*T*_g_ (K)	*T*_c_ (K)	*T*_m_ (K)
Spray drying	BCL–PVP 1:1 (SD)	375	-	-
	BCL–PVP 2:1 (SD)	357	416	444; 446
Evaporation	BCL–PVP 1:1 (E)	376	-	-
	BCL–PVP 2:1 (E)	357	396	449

**Table 2:** Particle size distribution results obtained using the laser diffraction method.

Process	Sample	Dv 10 (µm)	Dv 50 (µm)	Dv 90 (µm)
Spray drying	BCL–PVP 1:1 (SD)	3.6	16.4	34.9
	BCL–PVP 2:1 (SD)	4.6	17.1	60.1
	BCL (SD)	5.3	15.0	38.5
Evaporation	BCL–PVP 1:1 (E)	80.0	352.0	1087.0
	BCL–PVP 2:1 (E)	54.1	252.0	672.0
	BCL (E)	14.5	55.1	251.0

**Table 3:** Values of intrinsic dissolution rates of crystalline bicalutamide, drug processed alone and binary systems containing bicalutamide and PVP obtained using the evaporation technique (E) and spray drying (SD).

Process	Sample	IDR (mg/cm^2^/min)
-	Raw BCL	0.0144
Spray drying	BCL-PVP 1:1 (SD)	0.0796
	BCL-PVP 2:1 (SD)	0.0411
	BCL (SD)	0.0204
Evaporation	BCL-PVP 1:1 (E)	0.0992
	BCL-PVP 2:1 (E)	0.0523
	BCL (E)	0.0131

**Table 4:** Employed mathematical models.

Function	Equation
First-order	$Q=k\cdot t+Q_{0}$
Korsmeyer–Peppas	$Q=k\cdot t^{n}$
Hixson–Crowell	$Q^{\frac{1}{3}}=k\cdot t+Q_{0}{}^{\frac{1}{3}}$

*n* is the release exponent; *Q*, percent of drug dissolved at time *t*; *Q*^0^, percent of drug dissolved at time *t* = 0; *k*, dissolution rate constant.

**Table 5:** Parameters, determination coefficients and statistical significance of dissolution kinetics models.

Formulation	MDT (min)	DE (%)	Mathematical Model		
			Zero Order	Korsmeyer–Peppas	Hixson–Crowell
			(Constant Release)	(Diffusion Based Release)	(Erosion Based Release)
Raw BCL (not processed)	19.55	5.58	*k* = 0.1037 *Q*_0_ = 2.4571 *r*^2^ = 0.9594 *p* = 0.0035	*k* = 1.0895 *n* = 0.5002 *r*^2^ = 0.9981 *p* < 0.0001	*k* = 0.0117 *Q*_0_ = 2.66 *r*^2^ = 0.9036 *p* = 0.0114
BCL (E)	20.65	22.39	*k* = 0.4455 *Q*_0_ = 8.9816 *r*^2^ = 0.9686 *p* = 0.0024 *	*k* = 3.8641 *n* = 0.5362 *r*^2^ = 0.9991 *p* < 0.0001 *	*k* = 0.0199 *Q*_0_ = 10.02 *r*^2^ = 0.9131 *p* = 0.0096 *
BCL (SD)	15.99	53.05	*k* = 0.8489 *Q*_0_ = 27.25 *r*^2^ = 0.9040 *p* = 0.0130	*k* = 12.2261 *n* = 0.4501 *r*^2^ = 0.9827 *p* = 0.0011 *	*k* = 0.0216 *Q*_0_ = 28.13 *r*^2^ = 0.8440 *p* = 0.0263
BCL-PVP 1:1 (SD)	6.28	70.30	*k* = 0.4905 *Q*_0_ = 55.90 *r*^2^ = 0.5796 *p* = 0.1350	*k* = 35.4591 *n* = 0.2164 *r*^2^ = 0.8413 *p* = 0.0358	*k* = 0.0104 *Q*_0_ = 55.06 *r*^2^ = 0.5611 *p* = 0.1540
BCL-PVP 2:1 (SD)	3.60	83.93	*k* = 0.1530 *Q*_0_ = 82.11 *r*^2^ = 0.5043 *p* = 0.1790	*k* = 73.9170 *n* = 0.0512 *r*^2^ = 0.7916 *p* = 0.0464	*k* = 0.0027 *Q*_0_ = 82.02 *r*^2^ = 0.5014 *p* = 0.1840
BCL-PVP 1:1 (E)	7.65	73.17	*k* = 0.5099 *Q*_0_ = 58.37 *r*^2^ = 0.4847 *p* = 0.1920	*k* = 35.4333 *n* = 0.2287 *r*^2^ = 0.7469 *p* = 0.0769	*k* = 0.0107 *Q*_0_ = 56.933 *r*^2^ = 0.4759 *p* = 0.2100
BCL-PVP 2:1 (E)	5.19	79.11	*k* = 0.2097 *Q*_0_ = 75.53 *r*^2^ = 0.8494 *p* = 0.0260	*k* = 67.22 *n* = 0.0632 *r*^2^ = 0.9917 *p* = 0.0003 *	*k* = 0.0037 *Q*_0_ = 75.55 *r*^2^ = 0.8394 *p* = 0.0284

*r*^2^, determination coefficient; MDT, mean dissolution time; DE, dissolution efficiency; *k*, dissolution rate constant; *Q*_0_, percent of drug dissolved at time *t* = 0; n is the release exponent; *p* is the probability of ANOVA test for similarity (* <0.01).
